# A data processing approach with built-in spatial resolution reduction methods to construct energy system models

**DOI:** 10.12688/openreseurope.13420.1

**Published:** 2021-04-19

**Authors:** Christian Etienne Fleischer

**Affiliations:** 1 Department of Energy and Environmental Management, Europa-Universität Flensburg, Flensburg, 24943, Germany

**Keywords:** data processing, energy system modelling, spatial aggregation, sector-coupling

## Abstract

**Introduction: **Data processing is a crucial step in energy system modelling which prepares input data from various sources into a format needed to formulate a model. Multiple open-source web-hosted databases offer pre-processed input data within the European context. However, the number of documented open-source data processing workflows that allow for the construction of energy system models with specified spatial resolution reduction methods is still limited.

**Methods: **This paper presents a novel data processing approach to construct sector-coupled energy system models for European countries while maximising the use of existing web-hosted pre-processed data. Three power and heat optimisation models of Germany were constructed using different spatial resolution reduction methods.

**Results: **Significant variation in generation, transmission and storage capacity of electricity were observed between the optimisation results of the energy system models. The results of the model that used administrative state boundaries to define regions were found to be sensitive to the omission of solar rooftop photovoltaic availability.

**Conclusions: **This paper uses the proposed data processing approach to demonstrate the importance of spatial context when building and analysing power and heat optimisation models.

## Introduction

Since the early 2000s, energy system models are becoming more openly available. Before that, these models were primarily closed and proprietary. Maruf *et al.* identified 59 freely available energy system modelling tools ^1^. Energy system models are considered ‘open’ when the data and model code is accessible and legally usable ^2^. Pfenninger *et al.* discuss how open models improve the scientific quality of the models by adhering to fundamental scientific principles such as transparency and reproducibility ^3^. Pfenninger *et al.* also state that when models and data are open, productivity increases as it reduces the time spent by researchers in duplication of work in developing models and datasets ^3^.

The steps in the open-source energy modelling process are described by Pfenninger *et al.* in 2. One crucial step in that process is data processing. Data processing is an intermediate step between the raw input data and the model formulation. The input data is made accessible to the formulated model after undergoing data processing. The methods used to process the input data can have an impact on model results. Two documented impacts are the effects of temporal resolution reduction methods ^4– 9^ and spatial resolution reductions methods ^10– 14^. Therefore, the data processing steps must be well documented to ensure that their impact on the modelling results can be properly gauged. There are a limited amount of available open-source modelling tools and datasets that allow for the alteration of the spatial resolution of energy system models. One of these tools is presented in 15 by Hörsch *et al.*, which builds a highly spatially disaggregated European power system model dataset. The resolution of the dataset can then be reduced at various spatial scale by clustering the electrical network using the k-means algorithm. Tröndle *et al.* investigate the possibility of renewable energy autarky at various spatial scales using European power system models at three different spatial scales: continental level, national level, and regional level ^16^.

Input data of high spatial and temporal resolution can be generated using tools such as the global Renewable Energy atlas (REatlas) atlas ^17^, the Python Generator of renewable time series and maps (PyGreta) ^18^, and the GlobalEnergyGIS ^19^. The input data can also be obtained from an extensive list of web-hosted platforms, repositories and databases. These platforms and datasets include the renewables.ninja platform ^20^, the Open Power System Data (OPSD) platform ^21^, the hotmaps repository ^22^ and the ENSPRESO database ^23^. The Open Energy Platform compliments these platforms by documenting and sharing datasets used by existing energy system models such as the eTraGo ^24^, OSeMBE ^25^ and MEDEAS ^26^. These platforms facilitate the identification of documented and validated input data in centralised locations.

This paper presents a novel data processing approach that maximises the use of the web-hosted pre-processed input data to build energy system models. The data processing approach is used to build and compare power and heat models that have different spatial context.

## Methods

### Data processing workflow approach

**Figure 1.**
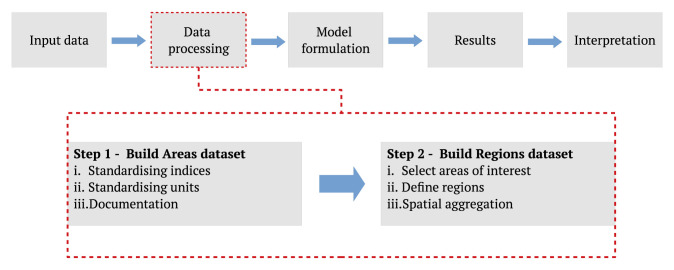
An illustrated description of the proposed data processing workflow of the energy system modelling process. The energy system modelling process is based on the Openmod Philosophy laid out by Pfenninger *et al.* in 2.

The proposed data processing approach can be split into two steps as illustrated in Figure 1. The first step builds the Areas dataset, which host the necessary data variables from the pre-processed input data sources within a structured framework. A set of requirements are defined for the Areas dataset to ensure standardisation and proper documentation of the data variables. The first requirement prescribes that the data variables need to be indexed using standardised reference keys. These reference keys allow the data to be uniformly organised according to spatial, temporal, and technological specifications. The Nomenclature of Territorial Units for Statistics level 2 (NUTS 2) and NUTS level 0 are the two spatial reference keys used by the Areas dataset to structure data with a spatial dimension. The second requirement ensures the use of standardised units of measurements. The standardisation of units ensures uniformity, allowing the use of the dataset for modelling without additional unit conversions. The final requirement for building an Areas dataset is the documentation of the data variables in the dataset. The documentation entails providing the source of the data variables and a description of the unit. 

Building a Regions dataset is the second step of the data processing process. The Regions dataset is constructed by first defining the areas of interest. The areas of interest could include all areas in the Areas dataset, or it could consist of a subset of areas. As the name of the dataset indicates, the Regions dataset allows for the grouping of areas into regions. The Regions dataset is described in more detail in *Regions dataset*.

***Dataset framework.*** Two requirements were defined to guide the selection process of the dataset framework used as the skeleton for the Areas and the Regions dataset. The first requirement is that the dataset framework should be able to handle data with more than one reference key. The need for multiple reference keys handling capability allows data variables to be referenced according to spatial, temporal, or even technological indices. The second requirement is that the dataset framework must integrate well with existing open-source energy modelling software and scientific analysis software. As there is a multitude of these software ^15, 27– 30^ written in the Python programming language ^31^, the dataset framework should be a Python-based software. The xarray dataset object in the xarray toolkit ^32^ was selected to build the datasets based on these two requirements.

There are additional benefits of using xarray to construct the datasets, as listed below:

- the dataset can be exported as a unidata network common (nc) file format that can be compressed to lower file sizes which eases sharing of the datasets;
- the dataset framework allows for documentation of the data variables.

As the datasets created can be stored and shared using a single file, they can also be made accessible using online archives such as Zenodo. Zenodo attaches Digital Object Identifiers (DOIs), which allows for the citation of the data. Hörsch *et al.* ^15^ and Tröndle *et al.* ^16^ both share different versions of their model on Zenodo using the nc file format.

***Areas dataset.*** The Areas dataset spatial scope includes the EU 27 countries with the exclusion of Cyprus and Malta and the addition of Norway, Great Britain, and Switzerland. The data variables in the dataset can be sub-divided into two sets, the base variables and the derived variables. The base variables are used to determine the derived variables.

The Areas and the Regions datasets have a total of seven reference keys. There are two spatial reference keys, namely *NUTS 0* and *NUTS 2*. Data variables using the *NUTS 0* reference key apply at the national level, whereas the *NUTS 2* level data are, for most countries, spatial administrative areas within the countries. There are four technology-specific reference keys: *techs*, *fuels*, *techs hydro,* and *techs hydro subset*. The *techs* and *fuels* reference keys reference the different technology types of the power plants variable. The *techs hydro* reference key is used to reference the technologies used to generate power from hydropower. The *techs hydro subset* dimension refers to a subset of the *techs hydro* reference key of hydropower technologies that have reservoirs to store water. The *time* reference key is used to index the time dimension of data variables. The *time* indices are the hourly intervals of a single representative year. The variables reference key, unit and sources are summarised in Table 1. Except for temperature and offshore wind capacity factor, the data variables in the Areas dataset are organised at the NUTS 2 spatial level. Therefore, the Areas dataset can be considered as a collection of data variables of 270 NUTS 2 areas.

**Table 1.** Summary of the data variables in the Areas dataset.

Data variable	Base/Derived variables	Reference key	Unit	Sources
NUTS 2 id	Base	*NUTS 2*	NA	33
Geometry	Base	*NUTS 2*	NA	33
Country code	Base	*NUTS 2*	NA	33
Temperature	Base	*time, NUTS 0*	Degrees Celsius	20
Population	Base	*NUTS 2*	people	34
Power plants	Base	*techs, fuels, NUTS 2*	MW	35– 37
Hydropower plants dispatch	Base	*techs hydro, NUTS 2*	MW	38
Hydropower plants storage	Base	*techs hydro subset, NUTS 2*	MW, MWh	38
Rooftop solar PV area	Base	*NUTS 2*	km ^2^	39– 42
Ground-mounted solar PV	Base	*NUTS 2*	km ^2^	39– 42
Onshore wind area	Base	*NUTS 2*	km ^2^	22, 43, 44
Offshore wind area	Base	*NUTS 2*	km2	43, 45
Solar capacity factor	Base	*time, NUTS 2*	Per unit capacity	20
Wind capacity factor	Base	*time, NUTS 2*	Per unit capacity	20
Offshore wind capacity factor	Base	*time, NUTS 0*	Per unit capacity	20
Hydropower capacity factor	Base	*time, NUTS 2*	Per unit capacity	46
Power	Derived	*time, NUTS 2*	MW	47
Heat	Derived	*time, NUTS 2*	MW	22
Air-sourced capacity factor	Derived	*time, NUTS 2*	Per unit capacity	20, 22

**Base variables**

There are a set of base variables needed to establish the foundation of the dataset. The identification code (NUTS 2 id), the geometrical information (Geometry), and the country identification code (Country code) of the NUTS 2 areas are base variables taken from the Eurostat database ^33^. The NUTS 2 geometry scale is 1:10 million. The ambient air temperature obtained from annual hourly historical weather data from the renewables.ninja platform ^20^ is a base variable in the NUS T 2 area dataset. The weather data is only available at the country level, so the temperature data is given at NUTS 0 in the dataset. The population values in the NUTS 2 areas are extracted from the Global Human Settlement (GHS) population grid ^34^. The population raster for the year 2015 was used. The population values within the geometry of the NUTS 2 area are summed, and the sum is the population value assigned to the NUTS 2 area. Other base variables describe the energy generation and storage technologies.

The power plants data variable gives the aggregated installed capacity of conventional power plants, solar photovoltaic (PV) installations, onshore and offshore wind installations associated with the NUTS 2 areas. Information on conventional power plants is from the conventional power plants dataset hosted on the OPSD platform ^34^. Information on solar and onshore wind installations for NUTS 2 areas in Germany, Denmark, France, Poland, United Kingdom and Switzerland, were extracted from the OPSD renewable power plants dataset ^35^. A power plant from the two datasets is associated with a NUTS 2 area when the power plants’ geometrical information places it within the geometrical boundaries of that NUTS 2 area. The power plants are grouped by the fuel and technology type and aggregated by their installed generating capacity in megawatts (MW). As the offshore wind installations are not within the geometries of the NUTS 2 areas, they are determined separately. The existing offshore wind installations were extracted from the European Marine Observation and Data Network (EMODnet) offshore wind farm database ^36^. This database provides the location of each wind farm as a georeferenced point and references it to a country. The offshore wind farms were assigned to the closest NUTS 2 area of the country it was referenced too.

The dataset has three categories of hydropower technologies: run-of-river hydropower, reservoir-based hydropower and pumped storage hydropower obtained from the Joint Research Council (JRC) hydropower plants database ^38^. The NUTS 2 areas were assigned the cumulative installed capacity of the different hydropower capacities. The reservoir-based and pumped storage hydropower plants’ cumulative storage capacity within the NUTS 2 areas was also calculated and added to the dataset. In the instances where the storage capacity was not given, it was assumed that the plant had a reservoir that can store the water needed to operate the plant at nominal capacity for six hours.

The availability of onshore wind and solar were assigned to the dataset as hourly capacity factor values extracted for each NUTS 2 area from renewables.ninja platform ^20^. The capacity factors for offshore wind at the national level was taken from the same data platform. The country-wide daily inflow from 46 defines the capacity factor of the hydropower plants provides the historical daily inflow in 30 European countries between 2003 to 2012. The hydropower plants’ capacity factors were calculated by dividing the daily inflow values by the sum of installed hydropower capacity within the country.

The data variables that define the area available for renewable energy technologies are rooftop solar PV area, ground-mounted solar PV area, onshore wind area and offshore wind area. For the NUTS 2 areas in the EU-27 countries and the United Kingdom, the ENSPRESSO database was used to assign the data variables of rooftop and ground-mounted solar PV area ^39^ and the data variables for onshore and offshore wind area ^43^. The areas classified in the EU-wide low restrictions with 400 m setback distance scenario were selected to define the onshore wind areas. The onshore wind areas in NUTS2 areas of Switzerland were calculated from the wind energy potential areas raster provided by the swiss energy ministry ^44^. The onshore wind areas in NUTS 2 areas in Norway were calculated from the wind energy potential areas raster of the hotmaps project ^22^.

The areas classified within the EU-wide low restrictions with water depth 0 – 30 m and water depth 30 – 60 m scenario were selected from 43 to determine the offshore wind area. Except for the NUTS 2 areas in Norway, the portion of the offshore wind areas assigned to a NUTS 2 area is proportional to their share of their respective country’s total coastline. The NUTS 2 areas of Norway are assigned offshore wind areas according to their proximity to the offshore wind areas listed in the Norwegian offshore wind strategic environmental assessment report ^45^.

The rooftop and ground-mounted solar PV areas in Switzerland and Norway were calculated using the Open Street Map building footprint data ^42^ and literature values. For Switzerland, the rooftop solar PV area in a NUTS 2 area was proportional to their share of the total building footprint area in Switzerland multiplied by a total available rooftop area of 267 km ^2^ and a rooftop suitability factor of 0.564 provided by Walch *et al.* in 40. For Norway, the rooftop solar PV area was calculated by multiplying the total building footprint area within the NUTS 2 areas with an rooftop area suitability factor of 0.49 calculated by Bódis *et al.* in 41. The ground-mounted solar PV area in Norway and Switzerland was calculated using the ratio of the ground-mounted solar PV area to the rooftop solar PV of Sweden and Austria, respectively. These ratios are 176:1 and 144:1, respectively.

**Derived variables**

Historical hourly electrical load profiles, at the country level, for European countries, are available on the European Network of Transmission System Operators (ENTSO-E) transparency platform ^47^. For the reference year selected, hourly load profiles of the NUTS 2 areas are multiplied with the NUTS 2 areas population factor. The population factor of a NUTS 2 area is the share of the population in that area in relation to the population of NUTS 2 area respective country.

Using a bottom-up approach, the heat demand profiles *D _a,t_* are generated for each NUST 2 area *a* and time step *t* using the following equation:

$$D_{a,t}=D_{a}\cdot \sum_{s,e}\left[\sigma_{a,s,e}\cdot d_{a,s,e,t}\right]\;(1)$$

The bottom-up approach classifies the heat demand in two end-use categories *e* and two sectors *s*. The end-use categories are space heating and domestic hot water heating. The sectors are the tertiary and domestic sector. Both end-use categories of each sector have a share factor σ *_a,s,e_* and a normalised hourly profile *d _a,s,e,t_* with time steps *t*. The share factor gives the percentage contribution of an end-use category of a sector to the total space and water heating demand. These share factors are country-specific and are obtained from the hotmaps repository ^22^. The hotmaps repository does not provide share factor values for Norway and Switzerland ^22^. Therefore, the share factors for Sweden and Luxemburg were used respectively instead. The normalised profiles are generated at the national level using generic profiles for space heating and water heating obtained from the hotmaps repository ^22^. The generic profiles for space heating are country, season and temperature-dependent whereas, the generic profiles of hot water heating only vary according to the day of the week and the season. The normalised space heating profiles are defined using the temperature data in the dataset. NUTS 2 areas within the same country are assigned the same normalised space heating demand profile. The heat demand volume *d _a_* for space heating and hot water heating is calculated from a rasterised map generated by the hotmaps project ^22^. The map depicts the estimated final energy demand for space and water heating on each hectare for EU28, Norway, Iceland and Switzerland for 2015.

The temperature variables from the dataset are used to calculate the hourly efficiency factors of the heat pumps. The efficiency of the air-sourced heat pumps is calculated using the Carnot coefficient of performance (COP) equation, a maximum COP value of 3.2 and a room temperature of 21°C.

***Regions dataset.*** Spatial resolution reduction is often used to reduce the computational demand of solving energy system optimisation problems. Depending on the research question or study focus, the data can be aggregated into regions to reduce the spatial resolution of the dataset. A common spatial resolution reduction method used by energy system modellers is to aggregate the spatial data according to political or administrative boundaries. European countries are classified according to multiple NUTS levels. The political regions method can thus group areas according to the NUTS level specified. For example, the spatial resolution of the data for Germany would reduce from 38 government regions of the NUTS 2 areas to the 16 states of NUTS 1. The spatial resolution could also be further reduced to a national level by aggregating the NUTS 2 area data to the NUTS 0 level. The number of NUTS areas at different levels is dependent on the European country. There are spatial resolution reduction methods that group areas according to the heterogeneity of spatial attributes of NUTS 2 areas. The max-p regions method for example, presented by Fleischer ^12^, groups areas into regions that are similar in population; wind and solar resource potential; and pumped-hydro storage capacity. The max-p regions method uses the max-p-regions algorithm, introduced by Duque *et al.* in 48.

Once the regions are defined, the variables of the NUTS 2 areas are aggregated to have variables that represent the regions. The resulting spatially aggregated dataset, hereafter referred to as the Regions dataset, is used to store and organise the variables generated after data aggregation. In the Regions dataset, the *NUTS 2* reference key is replaced by the *Regions* reference key. The *Regions* reference key is composed of the *NUTS 2* reference keys of the NUTS 2 area within the regions created. The geometry of the NUTS 2 areas attributed to the same region are joined to form the geometry of the regions.

The capacity factors of wind and solar are multiplied by a weight value before they are summed. The weight value is the share of land area value of the NUTS 2 area in relation to the land area in the respective appointed region. For the capacity factor of offshore wind, the weight value is the share of the offshore area in the NUTS 2 area in relation to the appointed region. The capacity factors of hydropower are weighed according to the area of the NUTS 2 areas in the regions. All other variables do not represent mean values and are summed without weight factors.

***Model formulation.*** There are some additional items needed in conjunction with a Regions dataset to formulate an energy system model. The first item is an energy system framework. There is a selection of open-source energy modelling frameworks that can be used. The selection of the framework depends on the focus of the study and the preference of the modeller. As the Regions database is generated using the python programming language, it can be integrated well into a python-based modelling framework.

Together with some additional items, the Regions dataset can then be used to formulate energy system models. One essential item is the techno-economic parameters. The techno-economic parameters will depend on the scenarios being investigated by the model. The scenarios also dictate certain assumptions used in the model.

### Power and heat optimisation model development

The proposed data processing workflow, implemented in the EUropean Sustainable Energy System (EU-SES) modelling tool ^49^, is used to build power and heat optimisation models to demonstrate the versatility of the data processing approach and the importance of spatial context in energy system modelling. The EU-SES tool uses the calliope framework ^27^ to formulate the models. The scripts used to generate the datasets, models and the optimisation results of each model can be found on Zenodo ^50^.

The first model is a multi-national model containing ten countries in the NUTS 2 area dataset named the GER NUTS0 model. These ten countries include Germany and nine countries that have a transmission connection with Germany. The NUTS 2 areas spatial data are aggregated according to national jurisdiction in the GER NUTS0 model. The second and third model reduces the spatial scope to include only Germany with no energy import or export from neighbouring countries. The difference between the second and third model is the spatial resolution reduction method used. The second model, named the GER NUTS1 model, are defined according to 16 administrative jurisdictions given by the NUTS 1 level. Whereas the regions in the third model, named the GER MAX-P model, are defined using the max-p regions method to generate nine regions. As illustrated in part a) of Figure 3, GER NUTS 1 model has more regions and therefore, the regions have, on average, a higher spatial resolution than the regions in the GER MAX-P model. The regions with the highest spatial resolution in the GER NUTS 1 model represent the city-states of Berlin, Hamburg and Bremen.

To investigate the effect of rooftop solar PV availability on the optimisation results of two models with the same spatial scope but with two different spatial resolutions, the GER NUTS1 and GER MAX-P model are optimised with and without the availability of rooftop solar PV areas.

**Figure 2.**
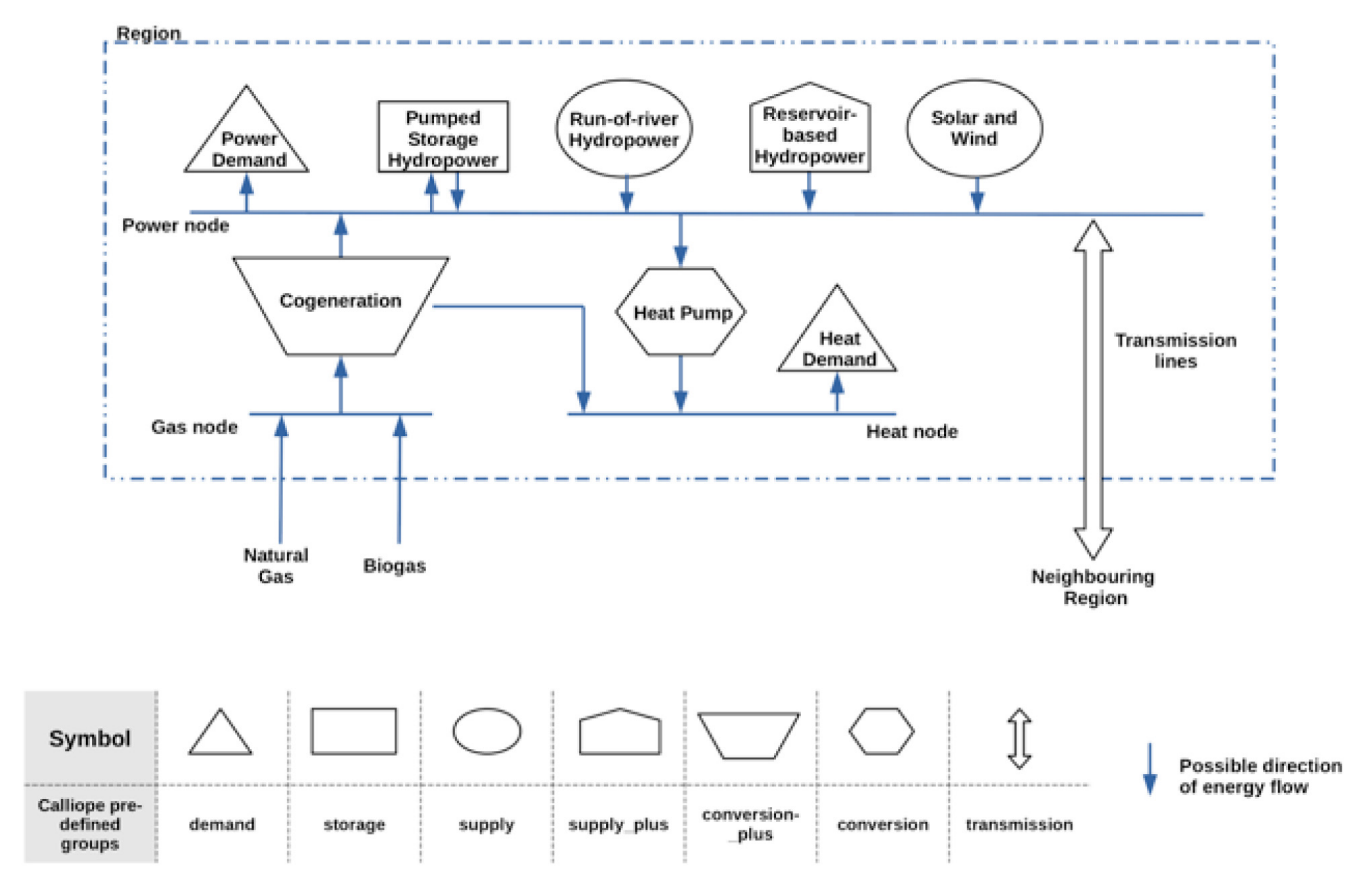
An illustrated description of the model created using the regions dataset and the calliope modelling framework. The table in the figure indicates the predefined technology groups used to describe the different components in the model.

The reference year selected to create the Areas dataset is 2010. The structure of the models is illustrated in Figure 2. The examples are modelled using the calliope modelling framework.

**Figure 3.**
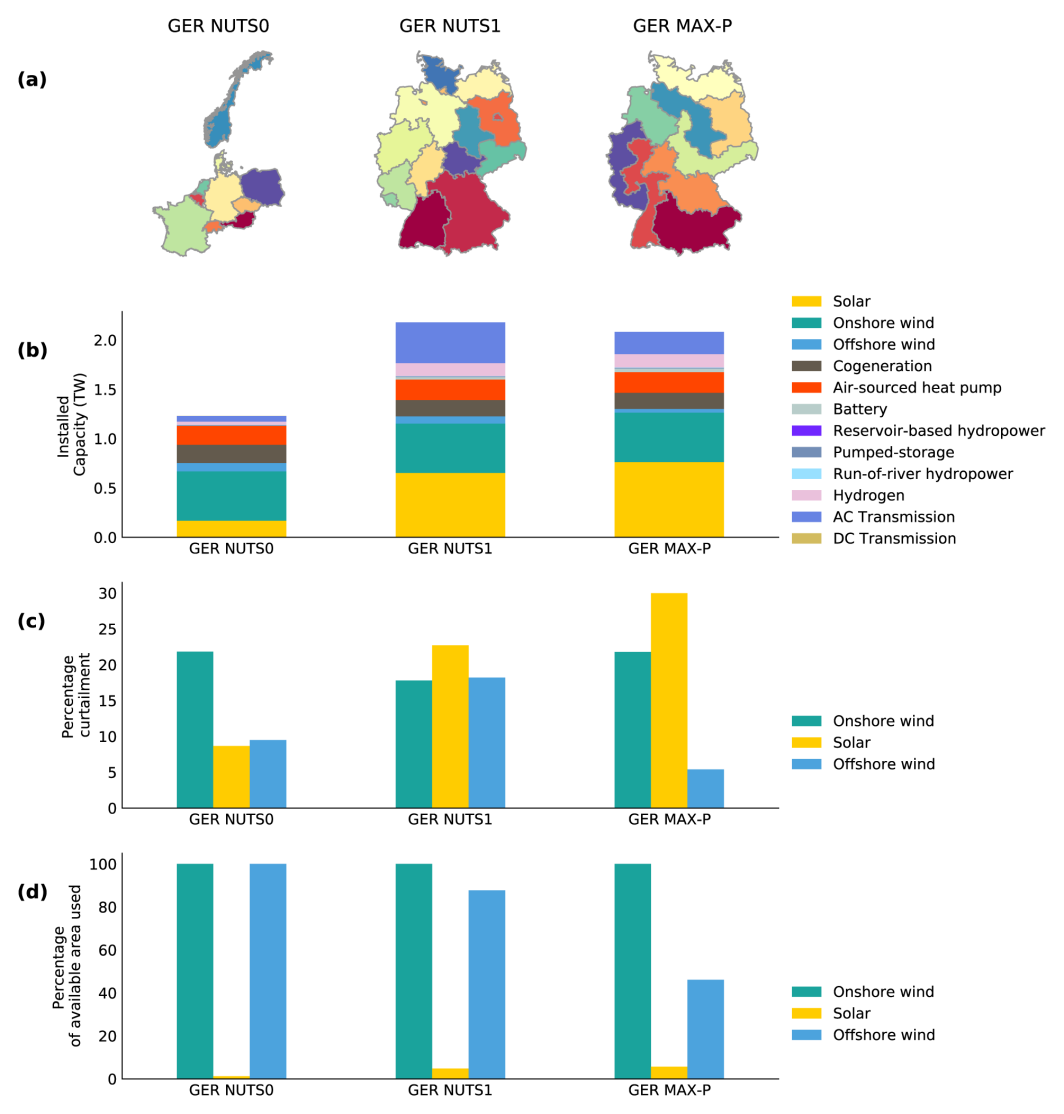
The regions in the GER NUTS0 model, GER NUTS1 model and the GER Max-P model, are illustrated in **a**). The least-cost optimisation results of the three models for Germany are given in **b**), **c**) and **d**). Plot **b**) illustrates the optimised installed capacity of the technologies. The curtailment rate of solar PV, onshore and offshore wind generation is given in percentage value in **c**). Plot **d**) illustrates the optimised percentage of the available area utilised by solar, onshore and offshore wind installations.

The models all share several overarching scenario assumptions. The following key assumptions are made in this scenario:

•   The cumulative CO _2 _equivalent emission of the optimised model is limited to 20 % of the 1990 CO _2 _equivalent emission of the countries in the model;

•   The cumulative biogas available to the cogeneration plants is 420 PJ which was estimated to be a projected value for 2020 presented by Scarlat *et al.* in 51;

•   All power plant capacities classified as biomass, gas and cogeneration in the Regions dataset are summed under the classification cogeneration;

•   Power plants classified as nuclear, coal, oil, other, waste and geothermal in the Regions dataset are not available in the model;

•   The storage level of all storage capacities is assumed to be full during the first and last instance of the optimisation;

•   The hydropower power plants, existing installed wind and solar capacities must be adopted in the optimised model;

•   All models have available only 50% of the ground-mounted area identified available for solar PV installations;

•   The solar and onshore wind capacity density is assumed to be 170 MW/km ^2^ and 5 MW/km ^2^, respectively, adopted from Ruiz *et al.* ^23^;

•   Offshore wind installations have a capacity density of 5.36 MW/km ^2^ adopted from Hundleby and Freeman ^52^;

•   Regions are considered ‘copper plates’, meaning that within the regions there are no constraints in energy transfer.

The power exchange between regions is possible and is constrained by the net transfer capacity and the efficiency of the power lines. There are two power transfer mediums in the model. The first is the high voltage alternative current (HVAC) transmission lines between regions that share a border. The HVAC has a minimum rated capacity of 2 GW and can be expanded by the model to a rated capacity of 8 gigawatts (GW). An HVAC transmission line capacity can be expanded at the cost of 400 €/MWkm adopted from Schlachtberger *et al.* in 53. The other power transfer mediums are direct current high voltage interconnectors installed between regions. The list of interconnectors and their respective rated transfer capacity is taken from the installed and planned DC links listed by Fleischer *et al.* in 12. Losses are not considered in the interconnectors and HVAC transmission lines.

The power and heat optimisation model’s objective function is to minimise the investment cost and dispatch cost of the model for one year and at a three-hour resolution. The optimisation models assume perfect foresight, and the power and heat demand is inelastic. A discount rate of 7% is assumed to calculate the annualised cost of the investments. The model uses techno-economic parameters projected for the year 2030, documented as *Extended data* ^54^. The techno-economic parameters for the generation and storage technologies were adopted from values presented by Moles *et al.* ^55^ and by Jülch ^56^, respectively.

## Results and discussions

The optimisation results of the three models are compared in Figure 3. The results in Figure 3 show that the GER NUTS 0 model has the lowest installed capacity of solar PV. This is despite the fact that Germany is represented at a lower resolution in the GER NUTS 0 model than the two other models and does not have the opportunity to maximise the use of good solar sites within Germany. This relatively low solar PV installed capacity of the GER NUTS0 model can be explained by the fact that the GER NUTS0 model has a greater spatial scope that the two other models. This additional benefit in spatial scope allows the GER NUTS0 model to maximise the use of resources available in neighbouring countries to Germany, such as hydropower-based energy storage capacities in Norway, Switzerland and Austria. These storage capacities can help minimise the curtailment rate of the solar PV installations, as illustrated in part c) of Figure 3. The lower-cost hydropower storage capacities in neighbouring countries can also explain why Germany in GER NUTS0 model does not invest in expensive storage technologies such as hydrogen. These apparent differences between the GER NUTS0 model and the models with a different spatial scope document the importance of spatial context in energy system modelling.

 Next, the optimisation results of the two models with the same spatial scope, the GER NUTS 1 model and the GER MAX-P model, are presented and discussed. The optimised transmission capacity of the GER NUTS 1 model is significantly greater than that of the GER MAX-P model, as can be seen in part b) of Figure 3, even though the installed capacity of the individual transmission lines is limited to a maximum of 8 GW. The fact that the GER NUTS 1 model has more regions, it can have more transmission lines, and therefore it can also have a higher optimised installed capacity value than the GER MAX-P model. The GER NUTS 1 model optimised solar PV installed capacity and optimised curtailment rate is lower than that of the GER MAX-P model. As shown in part c) of Figure 3, the optimised GER NUTS 1 model utilises almost half more offshore wind than the GER MAX-P model. The GER NUTS 1 model is limited in terms of space available for solar PV in city-states, with most of the space available for rooftop solar PV. This limitation in solar PV area combined with the transmission capacity expansion limits may have resulted in the more expensive offshore wind to become more economically viable. The differences between the two models that have the same spatial scope but constructed using two different spatial resolution reduction methods demonstrate the importance of spatial context in energy system modelling.

**Figure 4.**
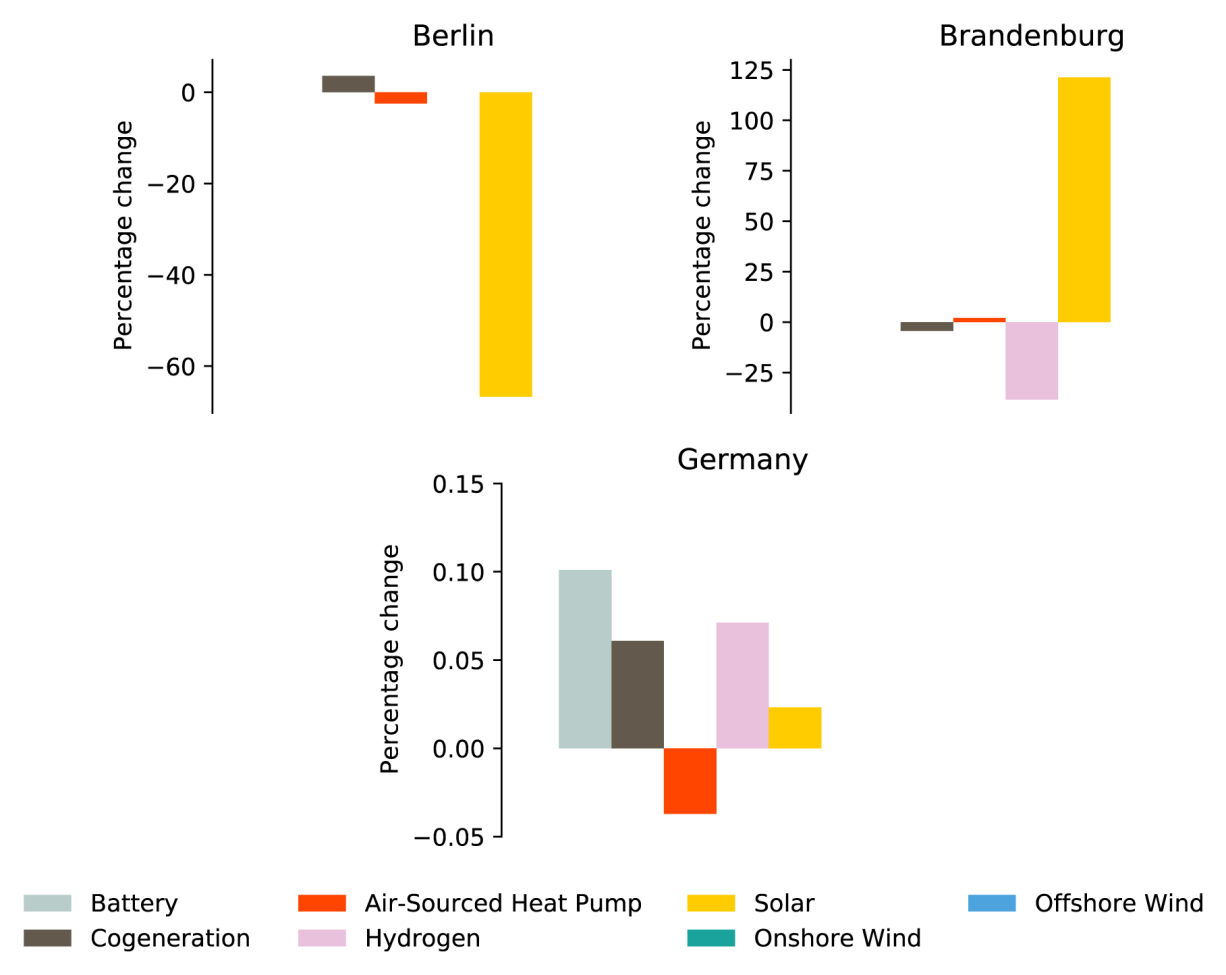
The three plots in the figure show the percentage change in installed capacity after the GER NUTS1 model was optimised without the availability of rooftop solar PV area. Only the technologies with a change in installed capacity are plotted. Two plots in the figure show the percentage change values for the two NUTS 1 region, Berlin and Brandenburg. The third plot shows the percentage change for the sum of installed capacities of all NUTS 1 regions in Germany.

 To investigate how the availability of solar PV can impact models with the same spatial scope but with different spatial resolutions, the GER NUTS1 model and the GER Max-P model was optimised without the availability of rooftop solar PV areas. There were no differences in the optimised GER Max-P model results with or without the availability of rooftop solar PV. Alternatively, the optimised GER NUTS1 model without rooftop solar PV did show some difference in the selection of technologies. These differences and changes in installed capacity are plotted in Figure 4 and Appendix 3. The city-state of Berlin is the NUTS 1 region with the most significant reduction of solar PV installed capacity after the rooftop solar PV areas were no longer available for solar PV installations. As seen in Figure 4, Berlin compensates for this loss of local electricity generation capacity with a relatively slight increase in cogeneration. The rise in cogeneration capacity impacts the adoption of air-sourced heat pump capacity, which sees a reduction in installed capacity. The removal of solar PV installations in Berlin seems to have an effect on neighbouring states such as Brandenburg, which sees a doubling in solar PV installation. Although the removal of rooftop solar PV area on the optimisation of GER NUTS1 model is significant for individual states such as Berlin, the national impact is minimal, as shown in Figure 4.

## Conclusion

A novel data processing workflow that maximises the use of the web-hosted validated pre-processed input data to build energy system models is presented. The proposed data processing workflow has a two-step process. The first step organises and standardises the pre-processed input data into a dataset called the Areas dataset. In the second step, the spatial data in the Areas dataset is aggregated according to regions and standardised into a Regions dataset. With the addition of techno-economic parameters and a modelling framework, the Regions dataset can be used to build power and heat models. The data processing approach is not integrated into any specific energy modelling framework, giving the modeller the flexibility to create a power and heat model using the modelling framework best suited for the research question. The proposed approach also provides a baseline that can be extended upon to include other energy sectors such as industry and transport. The proposed workflow is used to build three power and heat optimisation models. The three optimisation models' result reaffirms the importance of how the spatial scope and the method used for spatial resolution reduction can impact the optimisation result. By investigating the impact of rooftop solar PV availability on the optimisation results of two models that use two different spatial resolution methods, it was demonstrated that only the use of NUTS 1 state boundaries of Germany resulted in changes in optimisation results.

## Data availability

### Underlying data

Open Science Framework: A data processing approach with built-in spatial resolution reduction methods to construct energy system models. https://doi.org/10.17605/OSF.IO/D7JQY ^54^.

This project contains the following underlying data:

- Optimisation results (optimisation results of generated models saved as nc format files)
- Dataset (An areas dataset generated using the proposed data processing saved as nc format file)

### Extended data

Open Science Framework: A data processing approach with built-in spatial resolution reduction methods to construct energy system models. https://doi.org/10.17605/OSF.IO/D7JQY ^54^.

This project contains the following extended data in Extended data figures and tables.docx:

- Appendix 1 – Techno-economic parameters of generation technologies used the example models.
- Appendix 2 – Techno-economic parameters of storage technologies used the example models.
- Appendix 3 – Percentage difference in installed capacity of GER NUTS1 model without rooftop solar PV in reference to GER NUTS1 model with rooftop solar PV for all NUTS 1 administrative regions of Germany.

Data are available under the terms of the Creative Commons Zero “No rights reserved” data waiver (CC0 1.0 Public domain dedication).

## Software availability

- Source code available from: https://github.com/ENSYSTRA/EU-SES/tree/v1.2
- Archived source code at time of publication: https://doi.org/10.5281/zenodo.4646724 ^50^.
- License: Apache License 2.0 license.
