# Supplementary material for: A data processing approach with built-in spatial resolution reduction methods to construct energy system models
Source: Open Res Eur. 2022 Feb 10;1:36. Originally published 2021 Apr 19. [Version 2] doi: 10.12688/openreseurope.13420.2 (PMC10446009; doi:10.12688/openreseurope.13420.2)
Supplement: Peer review history

# Peer review: Reviewer response for version 2

**DOI:** 10.21956/openreseurope.15497.r28570

**Published:** 2022-03-03

**Authors:** Tim Tröndle

**Affiliations:** 1 Institute for Environmental Decisions, ETH Zürich, Zürich, Switzerland

I approve this revised version of the article.

 I have one remaining comment. I still think the introduction does not make the literature gap clear to the reader. It mentions existing tools but does not explain why they are insufficient or how this new approach expands their possibilities.

Reviewer Expertise:

Energy systems, open data.

I confirm that I have read this submission and believe that I have an appropriate level of expertise to confirm that it is of an acceptable scientific standard.

# Peer review: Reviewer response for version 2

**DOI:** 10.21956/openreseurope.15497.r28571

**Published:** 2022-03-03

**Authors:** William Usher

**Affiliations:** 1 Department of Energy Technology, KTH Royal Institute of Technology, Stockholm, Sweden

In my opinion, the revised article is ready for acceptance.

 I have spotted one small error - there is a missing reference on page 6 beginning the sentence "[] provides the historical daily inflow in 30 European countries..."

Reviewer Expertise:

energy system modelling; Python development; data processing workflows;

I confirm that I have read this submission and believe that I have an appropriate level of expertise to confirm that it is of an acceptable scientific standard.

# Peer review: Reviewer response for version 1

**DOI:** 10.21956/openreseurope.14490.r27282

**Published:** 2021-08-11

**Authors:** William Usher

**Affiliations:** 1 Department of Energy Technology, KTH Royal Institute of Technology, Stockholm, Sweden

**Overview**

 The paper presents a detailed description of the data processing required to conduct an analysis of European energy system. The data is aggregated in three different ways and the output from this process is then used to generate three alternative energy system models using the Calliope framework. The results differ between the models, although the explanation for these differences is insufficiently detailed.

 I believe this paper presents a useful addition to the research literature. In particular, the description of the data processing workflow is very detailed, providing clear guidance to others who wish to replicate or build upon this work. There is research-value in this data processing, but I would expect more critical reflection on the choice of data sources and implications of these choices. In addition, there is a good opportunity to investigate the modifiable areal unit problem (MAUP) and ecological fallacy – classical challenges in the aggregation of spatial data.

 At present, the data processing parts stops abruptly with no reflection on the process.

 The results and discussion almost exclusively focus on the results from the three models, but it is unclear in this section whether differences in the model results are derived from the choice of modelling framework, or from the aggregation of the data, or the interaction of the two (reviewer 1 highlights an example of this). The results seem rather weak or vague: “the transmission capacity expansion limits *may have* resulted in…” and “demonstrate the importance of spatial context in energy system modelling” and should be strengthened.

**Recommendations**

 I suggest that the authors refocus the paper on the data processing workflow, using an abbreviated version of the existing modelling results section as a case study to demonstrate the implications of different aggregation approaches.

 The introduction and results & discussion sections could be reformulated to highlight:

- The research gaps that the paper fills:
  - the need for high spatial and temporal analysis in energy.
  - there’s a lot of spatial data out there, but few papers which demonstrate how to bring it together.
  - how to avoid pitfalls when doing this.
- Whether existing data sets are sufficient, and where improvements are needed.
- What extra processing is required and implications of the assumptions and techniques used (such as for the derived variables).

The modelling case study should then reinforce some of these important points, for example demonstrating the response of the model to different aggregation methods.

**Other comments**

 Grammatical niggles: “Since the early 2000s, energy system models *have become* more openly available.” “There are a limited *number* of available open-source modelling tools…”

 The fact that the paper is unpinned with a Python package (EU-SES) to reproduce the results is not clearly highlighted separately from the energy system modelling. Clarifying the generalisability of the package would be useful e.g. how easy is it to use it with other energy system models? The following lines from the readme could be sufficient for this paper?

 import euses

 # First specify countries and year of interest

 # Use nomenclature of countries given in euses/parameters.py

 year = 2010

 countries = ['Austria','Switzerland']

 # The build_dataset function builds the xarray areas dataset

 example = euses.build_dataset(countries,year)

 example.create_regions('poli_regions')

 Derived variables: Discuss implications of electricity load profiles using population shares. E.g. errors introduced in low populated areas with high industrial demand?

 Could you show an example CO2 pathway of the cumulative cap of 20% of 1990 levels? How does this relate to policy?

 Results and discussions: “This is despite the fact that Germany is represented…” – use of “despite” seems incorrect, as I would expect a more aggregate model to omit detail on solar.

 As highlighted by reviewer 1, the rooftop solar result comes out of nowhere. If retaining this in the case study, it should follow from a research question.

 Figure 3d – clarify if % of available area used is absolute or relative to the 50% cap you impose – this is confusing to a reader.

Is the study design appropriate and does the work have academic merit?

Partly

Is the work clearly and accurately presented and does it cite the current literature?

Partly

If applicable, is the statistical analysis and its interpretation appropriate?

Not applicable

Are all the source data underlying the results available to ensure full reproducibility?

Yes

Are the conclusions drawn adequately supported by the results?

Partly

Are sufficient details of methods and analysis provided to allow replication by others?

Yes

Reviewer Expertise:

energy system modelling; Python development; data processing workflows;

I confirm that I have read this submission and believe that I have an appropriate level of expertise to confirm that it is of an acceptable scientific standard, however I have significant reservations, as outlined above.

# Response

**Published:** 2022-01-12

**Authors:** Christian Fleischer

**Affiliations:** Europa-Universität Flensburg, Germany

Author’s response to the review by William Usher **REPLY TO REVIEWER 2 COMMENTS** – (Reviewer’s comments in italics)

*I suggest that the authors refocus the paper on the data processing workflow, using an abbreviated version of the existing modelling results section as a case study to demonstrate the implications of different aggregation approaches.* *The introduction and results & discussion sections could be reformulated to highlight:*

- *The research gaps that the paper fills:*
  - *the need for high spatial and temporal analysis in energy.*
  - *there’s a lot of spatial data out there, but few papers which demonstrate how to bring it together.*
  - *how to avoid pitfalls when doing this.*
- *Whether existing data sets are sufficient, and where improvements are needed.*
- *What extra processing is required and implications of the assumptions and techniques used (such as for the derived variables).*

*The modelling case study should then reinforce some of these important points, for example demonstrating the response of the model to different aggregation methods.*

**Author reply: **Following recommendations from reviewer 1 and reviewer 2 to focus the article on the data processing workflow, the article type of the article has been changed to a Methods article. The method article describes the data processing and provides three case studies to demonstrate various ways that the workflow can be applied to build energy system models. The data gaps and what improvements are needed are now discussed in the results and discussions section. While addressing some of these recommendations the data processing approach was improved. The first improvement was the use of temperature data at NUTS 2 level to calculate the coefficient of performance of air-sourced heat. The data processing approach now allows technology specific proxy variables to be used when calculating the weighting factors. The proxy variables are given Table 2.  

*Grammatical niggles: “Since the early 2000s, energy system models have become more openly available.” “There are a limited number of available open-source modelling tools…”* **Author reply: **This phrase has been reformulated to – “In the past, energy system models were primarily closed and proprietary. However, recently more open-source energy system modelling tools have been made available.”  

*The fact that the paper is unpinned with a Python package (EU-SES) to reproduce the results is not clearly highlighted separately from the energy system modelling. Clarifying the generalisability of the package would be useful e.g. how easy is it to use it with other energy system models? The following lines from the readme could be sufficient for this paper?* *import euses* *# First specify countries and year of interest* *# Use nomenclature of countries given in euses/parameters.py* *year = 2010* *countries = ['Austria','Switzerland']* *# The build_dataset function builds the xarray areas dataset* *example = euses.build_dataset(countries,year)* *example.create_regions('poli_regions')”*

**Author reply: **A jupyter notebook, called *Three case studies*, was created to allow for the replication of the case studies and the results presented. The following sentence has been included in the “Power and heat optimisation model development” section to elaborate on the how easy is it to use it with other energy system models - The statement reads as, “The current version of the EU-SES tool can only automate the construction of an energy system model using the calliope framework. However, as the datasets are separated from models, the datasets can be used as input data in other modelling frameworks such as PyPSA.“ Also, to reduce the amount of time needed to solve the case study models the models were simplified in two ways. The first simplification is the removal of the battery technology in the model. The second simplification is the setting of the transmission capacity to a fixed value of 2GW and cannot be reduced or increased. The reference year used to build the Areas dataset for the case studies has been changed from 2010 to 2011.  

*Derived variables: Discuss implications of electricity load profiles using population shares. E.g. errors introduced in low populated areas with high industrial demand?*

**Author reply: **In the results and discussion section a few sentences are dedicated to address the limitations to using population data to disaggregate power profiles. Additionally, In the derived variable subsection, some references to studies that use population, GDP or a combination of population and GDP to disaggregate power profiles.     

*Could you show an example CO2 pathway of the cumulative cap of 20% of 1990 levels? How does this relate to policy?*

**Author reply: **To address this comment the following paragraph has been added to the article: “The cumulative CO _2 _emission constraint ensures that the models have high solar and wind penetration levels. This emission constraint aligns with the roadmap presented in 2011 by the European Commission that aims to reduce 80 % of the EU CO _2_ emission by 2050. In 2019 the EU commission revised the CO _2_ emission target for 2050 to a net-zero emission target ^10^. Therefore the 80 % reduction target could represent a snapshot along the net-zero pathway.  

*Results and discussions: “This is despite the fact that Germany is represented…” – use of “despite” seems incorrect, as I would expect a more aggregate model to omit detail on solar.* **Author reply: **With the change in article type the Results and discussion section has been edited to contain case studies that use the EU-SES modelling tool.  

*As highlighted by reviewer 1, the rooftop solar result comes out of nowhere. If retaining this in the case study, it should follow from a research question.*

**Author reply: **The models with 50% rooftop solar PV cap has been omitted as the article now focuses on describing the method and presenting some case studies.

*Figure 3d – clarify if % of available area used is absolute or relative to the 50% cap you impose – this is confusing to a reader.*

**Author reply: **The models with 50% rooftop solar PV cap were omitted therefore the available area is relative to the absolute available PV area.

# Peer review: Reviewer response for version 1

**DOI:** 10.21956/openreseurope.14490.r26739

**Published:** 2021-05-10

**Authors:** Tim Tröndle

**Affiliations:** 1 Institute for Environmental Decisions, ETH Zürich, Zürich, Switzerland

This work describes a data-processing pipeline which can be used to generate input data for models of the European energy system. The pipeline is used to build three models with different spatial scope and spatial resolution. All three models are then run twice: once with and once without the availability of rooftop photovoltaics. The main finding is that model results show a stronger change in one of the models.

 I believe the data-processing pipeline has the potential to be a valuable contribution to the scientific community but this article requires revisions. My main concerns are (1) mixing the data-processing pipeline with the actual energy system model, (2) the unclear research gap, (3) the conclusions, and (4) the relevance of the research question. I describe these points in more detail below.

 To me, the strongest part of this article is the data-processing pipeline. I suggest the author considers publishing it as a Data Note on this platform rather than a Research Article. Most of my points below regard the research part of this article and thus are invalid when published as a Data Note without the analysis.

1. The title, introduction, and large parts of the method section suggest that this article focuses on the data-processing step of energy system modelling: the pre-processing of raw input data that is necessary to build models. To test the impact of spatial resolution and scope, the author applies output of the data-processing step to energy system models and discusses model results. Mixing data-processing with the actual model obscures causal mechanisms and makes it difficult to attribute findings to their source. In fact, some of the findings are artefacts of the energy system models rather than the data-processing pipeline. For example, the author finds more transmission capacities in optimised systems with higher spatial resolution. This is due to a model decision of limiting the maximum installable transmission capacity independently of spatial resolution and it is therefore a model artefact rather than a consequence of the choice of spatial resolution of the data-processing pipeline.
  I suggest to make the impact of modelling choices on findings clearer in this article. In fact, I believe this article was stronger if it did not apply any energy system modelling but directly compared outputs of the data-processing pipeline.
2. The introduction mentions two existing data-processing approaches with the same purpose but does not mention any gaps in the existing approaches. I suggest to make it clear to the reader what the added benefit of this new approach is.
3. Modelling German city-states in isolation shows the largest sensitivity towards the lack of rooftop photovoltaics. This result is not surprising as the larger part of solar power stems from rooftops in a city-state like Berlin. I assume the model with lower spatial resolution leads to the exact same effect, but that effect is less visible as inputs and outputs are aggregated over larger areas. Is my assumption correct? If yes, what meaning do the findings have for modellers?
  I would like to verify this assumption myself, but I can not because model results are not published. Results from the models GER MAX-P and GER NUTS0 should be published in the same way results from GER NUTS1 are.
4. While not a criteria on this platform, I strongly recommend to improve the relevance of the research question. The study investigates the impact of a complete loss of rooftop solar potential in Germany using energy system models of different spatial scope and resolution. The relevance of this question and the choice of the analysis method is not clear to me. Why is this question relevant? What are the implications for further research?

Additional minor comments:

- Figure 2 seems to be incomplete, as the text mentions other storage options than the one shown. I suggest to add these options to the Figure or mention their omission in the caption.
- The JRC hydro database has been updated and improved several times since the reported access date. I suggest to use the most recent version in the pipeline as it resolves some of the problems mentioned.
- The study 16 has four spatial scales: continental, national, regional, and municipal.
- Zenodo can host datasets that consist of more than one file. The section “Dataset framework” seems to suggest the opposite.
- Some references in the section “Base variables“ point to the wrong source. For example, the OPSD platform is referenced with number 34, but the reference leads to an unrelated JRC publication.
- Some references need revision:
  - 6. Should cite the article rather than the pre-print.
  - 11. Should cite the article rather than the pre-print.
  - 16. Should cite the article rather than the dataset that is not used in this study.

Is the study design appropriate and does the work have academic merit?

Partly

Is the work clearly and accurately presented and does it cite the current literature?

Yes

If applicable, is the statistical analysis and its interpretation appropriate?

Not applicable

Are all the source data underlying the results available to ensure full reproducibility?

Yes

Are the conclusions drawn adequately supported by the results?

Partly

Are sufficient details of methods and analysis provided to allow replication by others?

Yes

Reviewer Expertise:

Energy systems, open data.

I confirm that I have read this submission and believe that I have an appropriate level of expertise to confirm that it is of an acceptable scientific standard, however I have significant reservations, as outlined above.

# Response

**Published:** 2022-01-12

**Authors:** Christian Fleischer

**Affiliations:** Europa-Universität Flensburg, Germany

Author’s response to the review by Tim Tröndle **REPLY TO REVIEWER** **1** **COMMENTS** – (Reviewer’s comments in italics)

*I believe the data-processing pipeline has the potential to be a valuable contribution to the scientific community but this article requires revisions. My main concerns are (1) mixing the data-processing pipeline with the actual energy system model, (2) the unclear research gap, (3) the conclusions, and (4) the relevance of the research question. I describe these points in more detail below.* *To me, the strongest part of this article is the data-processing pipeline. I suggest the author considers publishing it as a Data Note on this platform rather than a Research Article. Most of my points below regard the research part of this article and thus are invalid when published as a Data Note without the analysis.*

**Author reply: **As suggested the article type has been changed to focus the data processing pipeline. Although the reviewer suggested to change the article type to a Data Note type, following consultation with Open Research Europe editorial a Methods article type was found to best fit the content of the article. With the change in article type, the points by the reviewer that address the analysis are not addressed.  

*Figure 2 seems to be incomplete, as the text mentions other storage options than the one shown. I suggest to add these options to the Figure or mention their omission in the caption.* **Author reply: **The hydrogen storage option has been added to Figure 2. Note that the battery has been removed as a storage option in the models for simplification purposes and is therefore not included in Figure 2.  

*The JRC hydro database has been updated and improved several times since the reported access date. I suggest to use the most recent version in the pipeline as it resolves some of the problems mentioned.*

**Author reply: **The version of the JRC hydro database has been updated to V10 (most recent version on January 6 ^th^ 2021). V10 of the database still has the issue of not providing storage capacity of the hydropower plants with storage capabilities.  

*The study 16 has four spatial scales: continental, national, regional, and municipal.*

**Author reply: **The municipal spatial scale has been added to the article.  

*Zenodo can host datasets that consist of more than one file. The section “Dataset framework” seems to suggest the opposite.*

**Author reply: **To correct the suggestion made in this section the phrase has been reformulated to state, “The datasets created can be stored and shared using a single file in online archives such as Zenodo.“  

*Some references in the section “Base variables“ point to the wrong source. For example, the OPSD platform is referenced with number 34, but the reference leads to an unrelated JRC publication.*

**Author reply: **The reference for conventional power plants dataset hosted on the OPSD platform has been corrected with the correct reference number 37. Other misplaced references were not found in the Base Variable sub-section.  

*Some references need revision:* *- 6. Should cite the article rather than the pre-print.* *- 11. Should cite the article rather than the pre-print.* *- 16. Should cite the article rather than the dataset that is not used in this study.”*

**Author reply: **Suggested corrections have been made.
